# Primary and Hepatogenous Photosensitization in Livestock: A Review of Plant-Derived Phototoxins and Veterinary Implications

**DOI:** 10.3390/vetsci13030214

**Published:** 2026-02-25

**Authors:** Evelin Ramóna Péli, Dániel Cserhalmi

**Affiliations:** 1 Department of Botany, University of Veterinary Medicine, István u. 2, 1077 Budapest, Hungary; peli.evelin.ramona@univet.hu; 2 Rectorate for Research, Budapest University of Economics and Business, Markó u. 29-31, 1055 Budapest, Hungary

**Keywords:** furocoumarins, grazing livestock, hepatogenous photosensitization, hypericin, photosensitization, phototoxicity plant toxins, pyrrolizidine alkaloids

## Abstract

Photosensitization is a clinically significant dermatological and systemic disorder affecting grazing livestock worldwide. The condition arises following the ingestion or dermal exposure to photodynamic compounds that, upon activation by ultraviolet (UV) or visible light, induce tissue injury. Plant-associated photosensitization remains one of the most important aetiological categories in veterinary toxicology and may occur via primary (direct phototoxic) or secondary (hepatogenous) mechanisms. This review synthesizes current knowledge on the occurrence of photosensitizing compounds in plants, their biochemical and toxicodynamic properties, and their clinical relevance in livestock species. Emphasis is placed on major primary photosensitizing taxa, including *Heracleum* spp. and *Hypericum perforatum*, as well as hepatotoxic pyrrolizidine alkaloid-containing plants such as *Senecio* spp. Mechanistic pathways, plant metabolite ecology, and toxicopathological outcomes are discussed alongside illustrative case material. Plant-associated photosensitization also carries substantial economic consequences through decreased productivity, increased treatment costs and culling losses in affected herds and flocks. This condition represents a significant animal welfare concern in pasture-based production systems, where prolonged sunlight exposure, limited shade availability, and variable botanical composition of grazing areas can exacerbate both the incidence and severity of clinical manifestations. This review may provide a consolidated veterinary toxicology framework for understanding plant-associated photosensitization in grazing systems.

## Simple Summary

Plant-associated photosensitization is a significant health disorder in grazing livestock, arising after exposure to photodynamic compounds activated by ultraviolet or visible light. It may occur through primary phototoxic effects or secondary hepatogenous mechanisms. Key causative plants include *Heracleum* spp., *Hypericum perforatum*, and pyrrolizidine alkaloid-containing genera such as *Senecio*. This review outlines the biochemical basis, toxicodynamics, and veterinary relevance of these compounds, providing an integrated framework for understanding plant-induced photosensitization in grazing systems.

## 1. Introduction

Photosensitization reactions may be induced by numerous synthetic compounds, including antibiotics, antihistamines, anti-inflammatory agents, antimalarials, cardiovascular drugs, central nervous system-acting pharmaceuticals, and medications used in the management of multisystemic disorders [1], as well as by naturally occurring plant-derived bioactive substances [2]. For both synthetic and natural agents, a close relationship exists between chemical structure and photosensitizing potential. These compounds typically contain aromatic chromophore groups or photolabile functional bonds capable of absorbing light energy, generating free radicals, and initiating light-induced cellular damage.

Naturally occurring photosensitizing compounds found within certain plant families exhibit considerable diversity in chemical structure and biosynthetic origin. Their formation is linked to distinct metabolic pathways, and they may also be classified according to their modes of biological activity. Photosensitization may be triggered by exposure to plants in natural vegetation (e.g., grasslands) [3], cultivated agricultural crops [4], and species utilized in herbal medicine [5]. Consequently, their effects are relevant to human nutrition, animal feeding systems, and complementary or alternative medical applications.

Plant compounds responsible for photosensitizing effects in both animals and humans are secondary metabolites that play functional roles in plant physiology and ecology. Their concentration and distribution within plant tissues show substantial variability and are not governed by the regulatory dynamics of primary metabolic processes essential for basic survival. Instead, their quantitative expression is strongly influenced by biotic and abiotic environmental factors, as well as by diverse stress conditions [6].

Environmental variables that fundamentally affect plant growth conditions, cultivability, and productivity also modify the concentration and distribution of nutritional constituents and secondary metabolites within a given plant species [7]. In natural plant communities, such changes may additionally influence species distribution patterns within specific habitats [8]. Under current climate change projections, for example, periodic water scarcity is expected to alter vegetation structure and species composition. Such shifts may potentially increase the incidence of photosensitization-associated animal health disorders, both through conserved forage and during grazing.

Numerous secondary plant metabolites can cause dermal irritation without inducing true photosensitization (e.g., phorbol esters). The present review therefore focuses specifically on the mechanistic background of photosensitization, together with documented cases, causative agents, and plant taxa implicated in these reactions.

## 2. Role of Secondary Plant Metabolites

Whereas primary plant metabolites are universally essential for the maintenance of fundamental life processes and for the regulation of growth and development in all plant species [9,10,11,12], secondary plant products (SPP) or secondary metabolites play predominant roles in chemical signaling and in defense against grazing herbivores, insects, pathogens, and competing plant taxa [10,13,14]. Beyond their ecological functions, many secondary metabolites exert beneficial biological effects in both human and veterinary complementary medicine, including their utilization as medicinal plant products, nutritionally valuable feed components, or adjunct therapeutic agents.

The occurrence, concentration, and biological expression of secondary metabolites vary considerably among plant species and tissues. Owing to their extensive chemical diversity, these compounds may exert beneficial physiological effects. However, depending on their concentration and environmental context, they may also pose significant health risks.

SPPs synthesized by plants may act in grazing species as deterrents, irritants, or toxins. Nevertheless, behavioral avoidance mechanisms and physiological detoxification pathways in animals often mitigate the risk of irritation and overt poisoning [15]. A smaller subset of SPPs can induce photosensitization and associated toxicity in both humans and grazing herbivores, either through direct contact with plant tissues or following ingestion [16].

Without appropriate rangeland management, plant species containing toxic secondary plant products (SPPs) may expand and dominate larger grazing areas, thereby increasing the risk of toxicosis [17]. On the one hand, this may lead to feed avoidance and consequent production losses; on the other hand, certain phytochemicals may concurrently confer health-promoting effects in grazing animals [18]. Phytochemical concentrations also exhibit marked diurnal and seasonal variation. The levels of SPPs are generally lowest in autumn and highest during summer [19]; consequently, in pasture-based systems, the risk of photosensitization and other plant-associated toxicities is greatest during the peak grazing period.

## 3. Photosensitization Induced by Secondary Metabolites

In animals, photosensitization manifests clinically as severe dermatitis resulting from heightened reactivity of skin cells and tissues following exposure to sunlight. The condition develops when animals ingest or encounter plants containing photodynamic pigments (e.g., chlorophyll derivatives) or specific secondary metabolites such as naphthodianthrones and furocoumarins that are activated by ultraviolet (UV) or visible radiation [16,20]. Comparable reactions may also be triggered by photoreactive microbial or fungal metabolites [21,22].

Clinical lesions are typically most severe in non-pigmented, sparsely haired, or hairless skin regions [23]. Although the gross appearance may resemble solar dermatitis (sunburn), photosensitization represents a distinct pathophysiological entity. Its development is based on rapid photochemical reactions within skin cells triggered by both visible (400–700 nm) and UV-A and UV-B radiation, representing a broader excitation spectrum than that associated with classical sunburn.

Both spectral ranges can activate plant-derived phototoxins, initiating photosensitization. In contrast, sunburn lesions primarily result from excessive UV exposure, particularly within the UV-A range (315–400 nm), which penetrates deeper dermal layers and contributes to cumulative photodamage and premature skin aging [24].

During photosensitization, photo-excited oxidation initiates a cascade of reactions within the skin cells of affected animals, leading to direct injury of proliferative cell layers [16,23]. Photodynamic oxidation generates reactive oxygen species (ROS), including free radicals and singlet oxygen, through light-driven processes. In plant tissues, these reactive intermediates may induce DNA degradation [22,24].

In herbivorous animals, the pathological manifestation of these cellular events is photosensitization, reflecting the accumulation of plant-derived photodynamic molecules within target tissues and the circulatory system. Certain photoreactive compounds may also form DNA adducts through direct molecular interactions, thereby inducing photosensitization via oxygen-independent photochemical pathways [22].

Photosensitizing molecules are typically acquired during grazing and subsequently accumulate within the animal. Following systemic distribution or direct dermal contact, these compounds are localized in the skin, where they become activated upon exposure to sunlight. DNA damage arises through excitation induced by ultraviolet and/or visible radiation [24,25]. In addition to direct mechanisms, indirect photosensitization may develop secondary to hepatic dysfunction in grazing animals [22]. The difference between solar dermatitis and photosensitization is described in Table 1.

## 4. Mechanism of Action

Plants, as photoautotrophic organisms, derive energy through the absorption of light by specialized light-harvesting pigment systems. Within the thylakoid membranes of plant chloroplasts, the photosynthetic electron transport chain generates ATP and NADPH via sequential electron transfer reactions. These energy carriers are subsequently utilized in the Calvin cycle for carbon dioxide fixation and reduction [25].

During light absorption, only a proportion of pigment molecules enter an excited state capable of electron transfer. Among the most important excitable molecules are the photosynthetic pigments, particularly chlorophylls, which efficiently transfer photo-excited electrons to downstream acceptor molecules during the light-dependent phase of photosynthesis [22].

Environmental and physiological perturbations may, however, enhance photosynthetic electron flux and activity [26]. High light intensity represents a key example, leading to elevated production of reactive intermediates within plant tissues. Such conditions may simultaneously increase the photosensitizing potential of plants for grazing herbivores [23].

### 4.1. Photochemical Activation Process

In plants, photosensitization is primarily associated with chlorophylls and their derivatives, although analogous photochemical reactions may occur extra-plant via other reactive molecules [27].

The excited molecule (e.g., chlorophyll or its derivatives) is termed the photochemical activator (photosensitizer), whereas the molecule undergoing modification is referred to as the substrate or acceptor. All photochemical activators contain chromophore groups capable of absorbing light at specific wavelengths, thereby inducing intramolecular photochemical changes. These alterations subsequently affect associated biological substrates. When the photo-excited activator induces reactions involving molecular oxygen, the process is defined as photodynamic. Chlorophyll molecules located within intact chloroplast structures are normally protected from such photodynamic reactions [25,28]. In contrast, certain metabolites may become fully functional activators following metabolic transformation in acceptor tissues.

Some photoreactive agents (e.g., pyrrolizidine alkaloids) are relatively resistant to bacterial enzymatic degradation within the gastrointestinal tract and may therefore enter the systemic circulation in higher concentrations [27]. Other compounds, such as hypericin, are highly lipophilic, a property that facilitates their absorption and distribution within the body [29]. In contrast, chlorophyll-derived metabolites exhibit poor gastrointestinal absorption, limited binding and transport via glycoproteins, and lack the capacity to cross the blood–brain barrier [30]. Furocoumarins are known to be well absorbed in the gut [31]; thus, the chemical structure can determine the absorption rate of the toxins.

Activated plant metabolites induce photosensitization via two principal photochemical pathways, classified mechanistically as Type I and Type II reactions [27].

### 4.2. Photoreaction and Reactive Oxygen Species (ROS)

In Type I reactions, the excited activator interacts with the acceptor through electron transfer, generating radical ions in both molecules. Typically, the substrate donates an electron to the activator, producing a substrate cation radical (substrate^+^) and an activator anion radical (activator^−^), although the reverse may occur depending on the redox potentials of the interacting pair. In the presence of oxygen, both radical species may form oxygenated products, often resulting in oxidative consumption of the activator molecule. Alternatively, the extra electron of the activator anion radical may be transferred directly to molecular oxygen, generating superoxide radicals (O_2_^−^•) while regenerating the ground-state activator.

In Type II reactions, the excited activator transfers excess energy directly to ground-state molecular oxygen, converting it into highly reactive singlet oxygen while the activator itself returns to the ground state. Singlet oxygen subsequently reacts with biological substrates, producing oxidized metabolites. Unlike Type I reactions, the activator is not consumed but cycles between excited and ground states.

In vascular plants, singlet oxygen formation is intrinsically linked to energy transfer processes of photo-excited molecules [28]. Because chlorophylls function as natural photochemical activators, singlet oxygen generation is a ubiquitous phenomenon in plant tissues [32,33].

Beyond photosynthetic pigments, numerous secondary metabolites and phytotoxins produced by plant pathogens and microbes possess chromophoric structures capable of photochemical excitation. Photosensitizing secondary metabolites arise from diverse chemical classes, including: quinones, furocoumarins, polyacetylenes, thiophenes, benzofurans and chromenes. A shared functional property of these compounds is their ability to generate ROS upon photoactivation, particularly via Type II pathways leading to singlet oxygen production [28]. This capability is largely attributable to structural features such as polycyclic aromatic ring systems and conjugated double bonds. These chromophoric configurations enable efficient light absorption and intramolecular electron rearrangements, resulting in ROS formation, including singlet oxygen, superoxide radicals, hydrogen peroxide, and hydroxyl radicals [34,35].

The structural diversity, metabolic origins, and broad biogenetic distribution of plant photoreactive compounds suggest that these metabolites evolved multiple times independently during plant adaptive evolution. Their formation is closely linked to ecological defense strategies. Similarly, the evolution of ROS-mediated phototoxic mechanisms represents an important plant defense system against herbivores, pathogens, and pests [14,36].

### 4.3. Protective Metabolites

In addition to phototoxic compounds, plants also synthesize widely distributed metabolites that protect cellular structures from photo-oxidative damage. Notable examples include flavonoids possessing antioxidant activity, which stabilize membranes and cellular components against photodynamic injury [12]. A well-characterized compound is quercetin, a flavonoid derivative that functions as an efficient singlet oxygen quencher. Carotenoids provide further protection by directly quenching excited chlorophyll molecules and dissipating excess energy as heat, thereby limiting singlet oxygen formation [37]. Additional quenching agents present in plant membranes, including tocopherols, unsaturated fatty acids, specific proteins, water-soluble quenchers, comprise vitamin B6, ascorbate, various flavonoids [12,37]. Due to their photoprotective and antioxidant properties, plants rich in these compounds are widely utilized in the pharmaceutical and cosmetic industries. Their consumption or topical application is promoted for the protection of sensitive skin cells, mitigation of premature aging, and reduction in harmful effects associated with ultraviolet radiation exposure.

## 5. Occurrence of Photosensitizing Compounds in Plants

The formation of ROS in response to environmental stressors is a universal phenomenon across living organisms, including plants. During plant development, elevated ROS production has been documented under conditions of high light intensity, temperature extremes, and exposure to environmental pollutants [16,33,34]. Despite this, the photo-oxidative hazard posed by chlorophylls and their biosynthetic intermediates within intact plant tissues is considered negligible for grazing animals under normal circumstances [16]. In contrast, the biosynthesis of light-absorbing secondary plant metabolites is often maximized under increased UV-A and UV-B radiation, conditions that may promote the development of photosensitization following ingestion or contact [38].

In grazing livestock, the consumption of fresh green plant material represents the primary risk factor for the development of photosensitization when compared with hay or concentrate feeds [39]. This increased risk is largely attributable to the high concentrations of chlorophyll and protochlorophyllide molecules present in actively growing tissues, both of which may contribute to photodynamic reactions. Additionally, fresh plant material frequently contains higher levels of heterocyclic secondary metabolites capable of inducing photosensitization. In certain cases, combined exposure to plant pigments and secondary metabolites has resulted in enhanced phototoxic responses in grazing animals, suggesting potential synergistic interactions following co-ingestion [23]. Conversely, mechanical processing and preservation methods, including drying, chopping, haymaking, or ensiling, generally reduce phototoxicity and the likelihood of photosensitization after consumption. These observations are largely based on field and husbandry experience, as comparatively few studies have quantitatively analyzed differences in photosensitizing secondary metabolite content between fresh and processed forages. One earlier investigation compared phototoxic compound concentrations in processed versus fresh feed materials [40]. The study demonstrated that sun-drying resulted in an approximately 80% reduction in hypericin content in *Hypericum perforatum* (St. John’s wort), highlighting the substantial impact of post-harvest processing on phototoxic risk.

Comparative analyses of the chemical composition of photosensitizing agents across different plant organs may provide valuable insight into the pathogenesis of photosensitization in grazing animals. However, the precise influence of individual biotic and abiotic environmental factors on the biosynthesis and accumulation of plant photosensitizers remains insufficiently characterized. Elucidating these relationships represents an important area for future research, particularly with respect to biosynthetic pathways of photodynamic compounds, environmental regulation of secondary metabolite production, and tissue-specific accumulation patterns.

Understanding how global warming and climate change influence plant secondary metabolism—and specifically the production of photosensitizing compounds—is of growing veterinary and agricultural importance. Rising temperatures, intensified UV radiation, and drought stress are all projected to increase the concentration of secondary metabolites within green plant tissues [12,41]. Consequently, the incidence and severity of photosensitization, as well as broader plant-associated toxicities, may increase in grazing livestock populations [42].

Pre-processing of feed does not necessarily reduce the risk of photosensitization in livestock. For instance, the feeding of alfalfa silage has been reported to induce primary photosensitization in cattle, indicating that fermentation alone does not eliminate phototoxicity [43]. Secondary photosensitization may also occur when contaminated plant material is consumed in processed forms such as pellets, hay, or silage [44]. During storage, the concentration of photosensitizing compounds, particularly furocoumarins, may even increase under suboptimal conditions, for example, in mold-affected feed [45].

Importantly, toxicity is not determined solely by the degree of feed processing but largely by the molecular structure of the compounds involved. Furocoumarins are thermolabile and may degrade upon heating [46], whereas pyrrolizidine alkaloids remain structurally stable even at elevated temperatures [47]. Consequently, risk assessment must be conducted on a plant species–specific basis.

## 6. Development of Photosensitization in Animals

In susceptible animal skin, secondary plant metabolites are primarily responsible for the initiation of photosensitization reactions [22,25]. The integument of animals consists of three principal layers: the epidermis, dermis, and hypodermis (subcutis). The outermost layer of the epidermis is the stratum corneum (SC), which consists of keratinized, non-viable cells that are continuously desquamated and replenished by mitotically active cells originating from the stratum basale (SB) [48].

Damage to the epidermis, particularly the SC, compromises the protective barrier against environmental insults, including ultraviolet radiation and xenobiotic penetration. Consequently, deeper tissues within the dermis and hypodermis become more susceptible to both photonic injury and chemical insult. Cutaneous pigmentation also plays a protective role. Melanin present in darker skin absorbs and dissipates ultraviolet radiation, thereby reducing the risk of photosensitization [23].

When photosensitizing plant compounds are present, significant pathological changes may develop within susceptible skin tissues, including irritation, dermal necrosis, secondary bacterial infection, and severe trauma, potentially culminating in mortality [30]. In addition, endogenous amino acids within the skin, notably tryptophan, tyrosine, and histidine, are particularly susceptible to photo-oxidation. Their oxidation may trigger pronounced inflammatory responses within tissues and vasculature, contributing to necrosis of affected regions [16,26].

### 6.1. Routes of Exposure

Natural plant photosensitizers may reach the skin via two principal routes: direct plant–animal contact or systemic distribution via the bloodstream following ingestion [22,25]. These compounds exert cytotoxic (photocytotoxic) effects through direct cellular injury, or less commonly via immune-mediated (photoallergic) mechanisms.

The temporal onset of photocytotoxic photosensitization is highly variable. Clinical signs may develop (a) within minutes following direct dermal exposure, (b) several hours after systemic accumulation of primary photosensitizers, or (c) days later following activation of secondary photosensitizers (e.g., after hepatic injury and accumulation of phytoporphyrins in the skin). Photoallergic photosensitization may likewise manifest days after exposure via either contact or systemic pathways, although its precise pathogenesis in grazing livestock remains insufficiently characterized.

Following contact exposure, phototoxic compounds penetrate skin layers via two principal mechanisms. Polar compounds penetrate through active transport processes across the epidermis, while less polar (lipophilic) compounds diffuse passively through tissue layers [49]. Herbivore skin possesses several structural features that limit toxin penetration into sensitive subcutaneous tissues. The epidermis exhibits high resistance to the passive transport of bioactive molecules. The keratinized SC layer forms a substantial barrier to lipophilic compounds, which therefore tend to accumulate within this layer. This accumulation may further impede the diffusion of polar molecules into subepidermal tissues. Additionally, cutaneous lipids constitute an important physicochemical barrier. Removal or degradation of epidermal lipids increases dermal permeability to exogenous compounds [50].

Phototoxic compounds may also reach cutaneous tissues via systemic circulation following gastrointestinal absorption. This represents the primary exposure route in livestock. In ingestion-associated cases, tissue injury develops either through direct deposition of bioactive molecules in the skin or accumulation of secondary metabolites following hepatic dysfunction [13].

### 6.2. Host Susceptibility Factors

Sensitivity of grazing animals to dermally absorbed toxins and photosensitizers is influenced by multiple host and environmental factors, including species and breed, skin pigmentation, hair or wool density and thickness, age, general health status, ambient temperature, humidity, and precipitation [16]. Hair coats and skin pigments provide photoprotection by limiting ultraviolet penetration into epidermal layers. Consequently, lesions most commonly develop in sparsely haired or non-pigmented regions, including periocular areas, ears, facial regions and muzzle, mammary glands, perineal and tail regions, and coronary bands adjacent to the hoof wall [51]. Light-colored animals and those with thin hair or fleece are more susceptible than heavily pigmented or densely coated counterparts [52,53]. Species-specific histological differences in skin architecture may also influence susceptibility [49]. Young, debilitated, poorly pigmented, or hairless animals are generally more prone to cutaneous photosensitization than older, healthy, pigmented individuals.

### 6.3. Primary vs. Secondary (Hepatogenous) Photosensitization

Photosensitization in grazing animals is classified as either primary or secondary (hepatogenous) (Table 1) [23]. In primary photosensitization, phototoxic plant compounds—or their metabolites—become biologically active following ingestion or direct contact. After gastrointestinal absorption, they circulate systemically and accumulate within cutaneous tissues [16]. Primary photosensitization may also arise from abnormal porphyrin metabolism [48]. Its typically acute and rapid clinical course suggests direct intestinal absorption and swift dermal accumulation of photodynamic compounds, with limited time for secondary biochemical or cholestatic changes to develop [23]. Examples of primary photosensitizing agents include polycyclic compounds such as furocoumarin derivatives characteristic of the Apiaceae family and hypericin accumulating after ingestion of *Hypericum* spp. (Hypericaceae).

Secondary photosensitization results from systemic accumulation of the photodynamic phytoporphyrin called phylloerythrin. Phylloerythrin is a normal metabolic by-product of chlorophyll degradation during the digestion of green plant material in grazing animals. Following formation as an intermediate of chlorophyll catabolism, phylloerythrin is absorbed from the gastrointestinal tract (or rumen), enters systemic circulation, and is normally removed by the liver and excreted via bile.

When hepatic function is compromised—for example, due to hepatotoxic secondary plant metabolites—phylloerythrin clearance is impaired. Elevated circulating concentrations then accumulate within the skin, where photoactivation induces tissue injury. Although the precise phototoxic mechanism of phytoporphyrins remains incompletely defined, experimental evidence indicates localization within the Golgi apparatus and mitochondria, suggesting these organelles as primary cellular targets [54,55].

**Figure 1:**
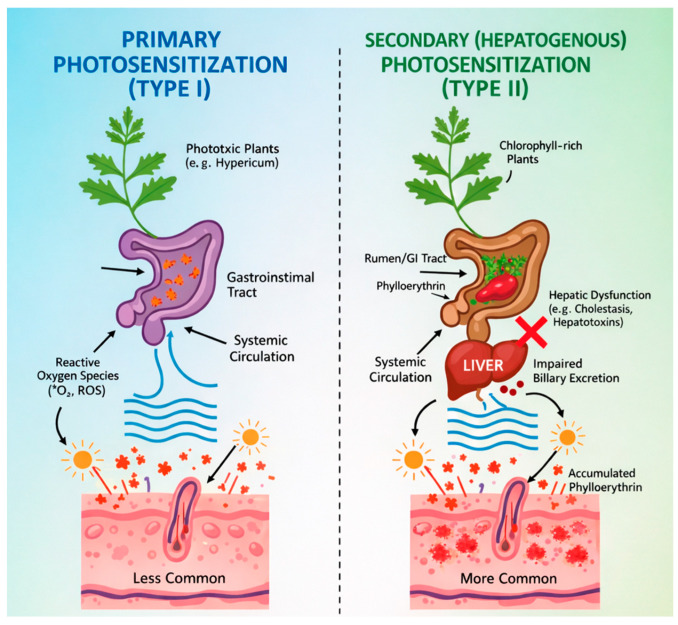
Comparison of primary and secondary (hepatogenous) photosensitization.

Secondary photosensitizers induce hepatic dysfunction through mechanisms including cholestasis (inhibition of bile flow into the duodenum) or direct hepatocellular injury. In such cases, porphyrins and their derivatives fail to undergo biliary excretion, leading to systemic retention and abnormal porphyrin metabolism [56]. Hepatogenous photosensitization therefore develops when ingestion of secondary plant metabolites results in liver disease or functional hepatic impairment [16]. Differences between primary and secondary photosensitization are presented in Figure 1.

Primary photosensitization occurs less frequently in grazing livestock than hepatogenous forms [23]. In addition to the plant taxa detailed in this review, numerous other species can induce both primary and secondary photosensitization syndromes [57,58,59]. However, only selected representative examples are discussed in detail in the present paper.

### 6.4. Clinical Pathology of Photosensitization

In primary photosensitization, early clinical signs include head shaking or tilting, restlessness, tail swishing, and active seeking of shade and water. Ocular involvement is common and may present as conjunctivitis, blepharitis, and edema of the eyelids and periocular tissues; drooping of the pinnae may also be observed. Severe bilateral periorbital and conjunctival edema, together with variable subcutaneous facial swelling, can develop in advanced cases. Icterus is not a feature of primary photosensitization. Serum biochemistry is typically unremarkable, and liver enzyme activities generally remain within reference intervals, although mild increases in gamma-glutamyl transferase (GGT) have occasionally been reported. Histopathological examination of affected skin may reveal necrotizing dermatitis and epidermal hyperplasia [60,61].

Hepatogenous photosensitization initially presents with similar cutaneous signs, including erythema of the muzzle and edematous periocular tissues. As the condition progresses, the muzzle may become covered with serous exudate, which subsequently dries to form thick, dark crusts and areas of sloughing. A key distinguishing clinical feature is the presence of systemic illness, characterized by depression and icterus.

Pathologically, the liver is often pale orange and friable, and may exhibit diffuse interlobular bile duct proliferation with marked cholestasis. Histologically, moderate to severe centrolobular degeneration and necrosis are evident, with enlarged and vacuolated hepatocytes. In contrast to primary photosensitization, serum biochemistry in hepatogenous cases typically demonstrates elevated aspartate aminotransferase (AST), increased GGT activity, and raised total bilirubin concentrations; creatinine may also be elevated in advanced cases [62], contributing to a high icteric index.

Reported morbidity ranges widely from 5% to 100%, depending on exposure conditions and herd management, while mortality is generally higher and more frequent in hepatogenous forms of the disease [63].

## 7. Plant Species and Compounds Causing Primary Photosensitization

Relatively few SPPs are classified as primary photosensitizing agents. This is partly attributable to the complex pathogenesis of photosensitization and to the methodological challenges associated with confirming compound-specific mechanisms of action and bioactive metabolite effects within animal tissues. Only a limited number of phytochemicals have been demonstrated to induce both primary and secondary (hepatogenous) photosensitization in grazing herbivores, whereas others are recognized exclusively as causative agents of secondary forms.

### 7.1. Hogweeds (Heracleum spp.)

Species of the genus *Heracleum* (family Apiaceae) are large herbaceous plants widely distributed across Europe. The three most prevalent species include *Heracleum sphondylium*, *Heracleum mantegazzianum* and *Heracleum sosnowskyi*. Common hogweed (*H. sphondylium*) is native to Europe and typically occurs in moist meadows, forest margins, woodland clearings, and roadside habitats, often associated with nitrogen-rich soils. Its toxicity is lower than that of the larger invasive congeners; however, dermatitis may still occur. When incorporated into forage or encountered through direct contact, it may induce clinical disease, particularly in cattle.

Each *Heracleum* species synthesizes both linear and angular furocoumarins; however, the relative proportions of these compounds vary among species. Phototoxic potency is influenced by the structural profile of these metabolites, with a higher proportion of angular furocoumarins generally associated with increased phototoxic activity. Consequently, *H. mantegazzianum* and *H. sosnowskyi* are considered particularly potent inducers of photosensitization due to their comparatively elevated content of angular furocoumarins [64]. The latter two species may reach heights of 3–5 m and are classified as invasive in many regions. Their distribution is sporadic but locally dense, and eradication programs are implemented in several European countries due to ecological and public health concerns. Giant hogweed (*H. mantegazzianum*) is regarded as one of the most significant invasive plant health hazards in Europe.

Contact with *H. mantegazzianum* followed by sunlight exposure induces severe cutaneous injury, clinically diagnosed as phototoxic dermatitis [65,66,67]. The plant is characterized by large, serrated leaves, hollow stems covered with fine bristles and dark purple blotches, and prominent compound umbels up to 80 cm in diameter composed of small white flowers [68]. Both living plants and cut plant material may trigger lesions. As a perennial species, giant hogweed poses substantial risks to both human and animal health via direct contact with photosensitizing sap, producing burns resembling thermal injury on sun-exposed skin [65,69]. The sap of *H. mantegazzianum* contains photosensitizing furocoumarin derivatives [68,70]. These metabolites, particularly angelicin, occur at highest concentrations in leaves but are also present in stems and inflorescences [71].

**Figure 2:**
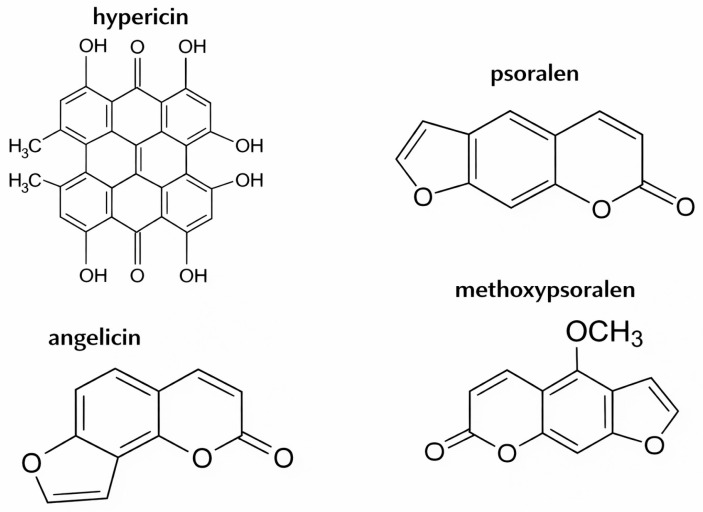
Major SPPs causing primary photosensitization.

Ecologically, furocoumarins confer adaptive advantages through insecticidal activity and antimicrobial effects, thereby protecting plants from herbivory and pathogen attack [72,73,74]. In mammalian cells, furocoumarins exert mutagenic and carcinogenic effects via direct DNA intercalation. Following integration, photoactivation induces covalent cross-linking of these tricyclic molecules. Subsequent reactions with pyrimidine bases on complementary DNA strands increase intra-strand cross-link formation [75,76,77]. These cross-links induce apoptosis and inhibit cellular replication. Key furocoumarins identified in *H. mantegazzianum* include angelicin, psoralen, and methoxypsoralen [68] (Figure 2). Severe dermal injury following sap contact is therefore attributable to profound disruption of cellular integrity within exposed skin tissues.

### 7.2. St John’s Wort (Hypericum perforatum)

*Hypericum perforatum* is a perennial species widely distributed throughout temperate regions. Numerous reports worldwide have documented primary photosensitization in livestock following grazing or ingestion of this plant [52,78,79]. The principal phototoxic compound is hypericin (Figure 2), a photocytotoxic secondary metabolite. Hypericin accumulates in specialized secretory glands within leaf tissues [37,40,80,81]. Threshold doses for photosensitization vary by species. For partially pigmented Hereford-cross cattle, it is estimated at about 10.5 mg/kg body weight, but it’s different for other breeds or species [53]. In sheep, clinical disease has been reported at approximately 3 mg/kg dry matter intake [27,52], whereas cattle exhibit comparatively higher tolerance [82]. Clinical manifestations correspond to classical UV-associated photosensitization, including erythema of the muzzle, periocular tissues, and ears, edema of exposed skin and elevated rectal temperature [78].

Hypericin is a phenanthroperylene quinone capable of forming strong complexes with diverse protein targets. Owing to its photodynamic properties, it remains under investigation for therapeutic applications, including photodynamic therapy [37,83]. Upon photoactivation, hypericin generates multiple reactive oxygen species, including singlet oxygen (^1^O_2_), superoxide anions, and hydrogen peroxide (H_2_O_2_) [84,85,86,87]. Each of these ROS can induce cell death in UV-A-exposed tissues.

Hypericin biosynthesis is enhanced under UV-B and full-spectrum UV exposure in both greenhouse-grown and field-grown plants. Longer-wavelength radiation may likewise promote reactive molecule formation in plants and grazing livestock. Because these wavelengths penetrate tissues more effectively, they contribute to increased photosensitivity and dermatitis severity [88].

Hypericin-induced ROS causes cellular injury and loss within the skin via apoptosis, necrosis, and possibly autophagy pathways. Tissue damage depends on the spatial co-localization of ROS generation and cellular target molecules, as ROS diffusion is limited due to their high reactivity and short half-life [89,90,91,92,93]. Hypericin binds preferentially to membranes of key intracellular organelles, including mitochondria, endoplasmic reticulum, Golgi apparatus, and lysosomes [87,91,94,95,96,97]. These structures are therefore considered primary subcellular targets of photodynamic injury. When photoactive compound accumulation coincides with light exposure, ROS-mediated degradation of proliferative cells within the SB ensues, producing clinically significant photosensitization in affected livestock. Removal of animals from pastures containing *Hypericum* spp. is typically sufficient to reduce systemic hypericin levels and mitigate further phototoxic reactions [52,68].

## 8. Plant Species Causing Secondary (Hepatogenous) Photosensitization and Their Compounds

Pyrrolizidine alkaloids (PAs), as secondary plant metabolites, induce significant hepatic injury in animals consuming plants containing high concentrations of these compounds [16]. These metabolites may lead to hepatogenous photosensitization, which develops secondary to toxic liver damage. Unlike primary photosensitizers such as hypericin, pyrrolizidine alkaloids do not exert direct effects on biliary excretory processes. Nevertheless, the severity of hepatic injury they induce may indirectly impair these pathways, ultimately resulting in photosensitization.

PAs occur across numerous plant genera; approximately 3% of all plant species are known to contain at least one PA compound, with nearly 350 structural forms identified to date [88,98,99]. Most plant species responsible for PA-associated hepatotoxicity in livestock belong predominantly to the families Boraginaceae and Asteraceae. The hepatotoxic and genotoxic potency of individual pyrrolizidine alkaloids varies considerably; however, many can induce severe acute and chronic hepatic insufficiency and dysfunction, frequently culminating in death.

PAs share a broadly similar mechanism of action in affected grazing animals. Following ingestion, they are metabolized within hepatocytes, particularly in the centrilobular (zone III) region of the liver. This region exhibits elevated enzymatic activity and lies furthest from the vascular supply, rendering it especially vulnerable to toxic injury. Centrilobular hepatocytes possess high concentrations of enzymes catalyzing oxidative, reductive, and hydrolytic reactions. These enzymatic systems participate both in toxin biotransformation and in the generation of biologically active secondary metabolites [100].

Structurally, PAs contain a pyrrolizidine nucleus that undergoes hepatic dehydrogenation, forming reactive pyrrolic metabolites, the principal cytotoxic derivatives of PAs [101]. The cytotoxicity of pyrroles arises from covalent binding to DNA and cross-link formation with proteins and amino acids. These interactions produce both direct cytotoxic effects and potent inhibition of cellular mitosis [98]. Due to their high reactivity, pyrrolic metabolites typically exert their toxic effects locally at the site of formation within hepatocytes.

Characteristic tissue-level lesions associated with PA hepatotoxicity include hepatocellular hypertrophy, hepatocellular necrosis, nuclear enlargement (megalocytosis), and progressive fibrosis [16,102]. DNA cross-linking disrupts normal mitotic processes, producing profound cellular dysplasia. Hepatocytes in PA-affected livers may become up to 30 times larger than normal, reflecting substantial DNA accumulation and failed cell division [98]. Severe hepatic dysfunction may culminate in death. Both acute and chronic liver injury can also precipitate photosensitization due to impaired hepatic metabolism and biliary excretion of phototoxic compounds, leading to their systemic accumulation.

Pyrrolizidine alkaloids (PAs) can induce both acute and chronic poisoning, although ruminants generally exhibit relatively low susceptibility to these toxins. Because PA concentrations in fodder are typically low and the distribution of PA-containing plants within pastures is often limited, acute intoxication occurs only rarely. However, certain PAs may persist in animal tissues and be released over time, making chronic poisoning more common in ruminants [103,104].

Interspecific differences in PA tolerance are also possible. Horses are highly sensitive to PA toxicity, whereas sheep, cattle, and goats demonstrate comparatively greater tolerance [103]. Nevertheless, other studies [105] indicate relevant differences even among ruminant species; therefore, a potentially higher tolerance in specific ruminants is plausible, but not yet fully validated.

### Ragworts (Senecio spp.)

Species of the genus *Senecio* (family Asteraceae) are widely distributed in natural ecosystems and represent a major toxicological concern for livestock producers, owing to their pronounced toxicity in ruminants and horses [16,51]. Pyrrolizidine alkaloids present in *Senecio* species are relatively resistant to microbial degradation within the gastrointestinal tract, permitting absorption into systemic circulation at higher concentrations. One determinant of toxicity variability lies in the marked structural diversity of alkaloid forms present among plant species.

**Figure 3:**
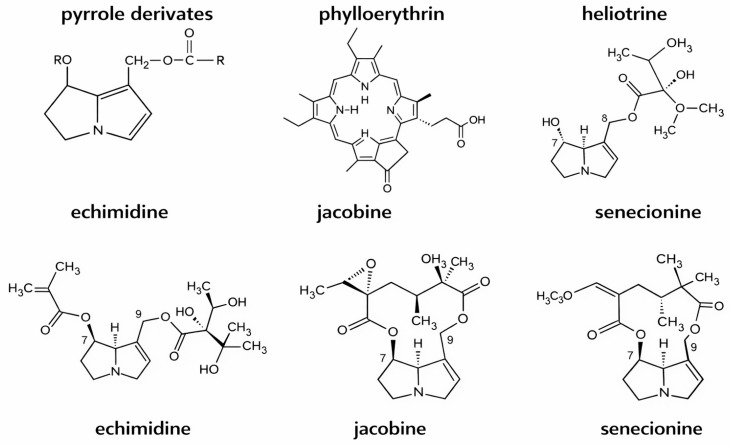
Major SPPs causing secondary photosensitization.

A principal bioactive PA in *Senecio* spp. is jacobine, which contains a macrocyclic (closed-chain) diester structure. This contrasts with the open-chain diester alkaloids, such as echimidine and heliotrine, found in genera including *Echium* and *Heliotropium*. Another common PA in *Senecio* species is senecionine, synthesized in the roots and translocated to aerial tissues during plant development (Figure 3). Macrocyclic (closed-chain) diester pyrrolizidine alkaloids exhibit substantially greater resistance to bacterial enzymatic degradation within the intestine. Consequently, they undergo enhanced metabolic activation to toxic pyrrolic intermediates within hepatocytes [99].

The cytotoxic hepatic effects of PAs impair the normal degradation and biliary excretion of phylloerythrin, a chlorophyll-derived photodynamic pigment. Its systemic accumulation leads to cutaneous photosensitization in affected animals. Importantly, pyrrolizidine alkaloids do not exert direct phototoxic effects within the skin of grazing herbivores, in contrast to primary photosensitizers such as hypericin. Instead, dermal photosensitivity develops secondarily to hepatic dysfunction and subsequent retention of circulating photodynamic metabolites [51].

## 9. Case Studies

In 2014, intoxication was reported in a flock of 27 Awassi sheep grazing on pasture heavily infested with *Hypericum perforatum*. Twelve animals exhibited only elevated body temperature, whereas 15 showed edematous swelling of the head, eyelids and ears, accompanied by dermatitis typical of photosensitization on unpigmented and non-wool-covered areas, primarily the limbs and udder. 12 animals died due to hyperthermia. Treatment consisted of corticosteroids, antihistamines and antibiotics, resulting in full clinical recovery [106].

Although grazing animals generally avoid *Hypericum* spp. under free-range conditions, outbreaks may occur under confined management. In a flock of 700 penned sheep, approximately 200 animals developed clinical signs within three days, as restricted feeding conditions enabled animals to graze the plants down to the stem base [107].

A recent survey further demonstrated that *Hypericum*-associated photosensitization occurs more frequently in sheep than in cattle. In a population-level assessment of 8200 animals, mortality among clinically affected individuals reached 14%. Clinical signs were most prevalent between May and September and included pruritus and cutaneous lesions and skin infections. In addition to systemic therapy, topical zinc oxide formulations were applied to support wound healing [108].

In all presented studies, animals grazed in extensive production systems, which increases the probability of photosensitization. When reported, the total mortality rate remained below 1%, which is consistent with the generally low mortality observed in the primary form of toxicosis [63]. Clinical signs were typical, as described in Section 6.4 [60,61]. Animals occasionally grazed stubble fields, which represent a consistently higher risk for exposure to potentially toxic agents [109].

On a mixed sheep and cattle farm, animals grazed freely on pasture heavily contaminated with *Senecio* species. In sheep, initial clinical signs included weight loss, apathy and photosensitization lesions affecting the ears and nasal region. Red blood tests revealed mild normocytic normochromic anemia. With progression of disease severity, euthanasia was required. Post-mortem examination revealed irregular hepatic architecture; in some cases, the liver appeared pale, firm and fibrotic, accompanied by gallbladder distension. Necropsy findings included hydropericardium, ascites, icterus, hydrothorax and mesenteric edema. Histopathology demonstrated marked degenerative changes, including hypertrophic Kupffer cells and hepatocellular enlargement (megalocytosis). Spongiform degeneration was also observed in cerebral tissues [110].

Poisoning was also associated with poor grassland quality. In one case, vegetation cover was sparse, whereas in another, the pasture was overgrazed. When available, the total mortality rate was below 1%, although mortality is generally higher in the hepatogenous form of photosensitization [63]. Mortality did not differ significantly between cattle and sheep, suggesting comparable susceptibility; however, a relatively higher tolerance in sheep remains plausible.

In cattle, chronic diarrhea, poor body condition and edema of the dewlap are also frequently reported. Hepatic lesions typically include reduced liver size, gray discoloration, and firm consistency, with fibrosis and bile duct hyperplasia evident on histological examination. Hepatocellular karyomegaly/megalocytosis was also relevant. Neuropathological lesions have been detected at the junction of gray and white matter [111].

*Senecio* toxicity is not restricted to ruminants. In horses, clinical manifestations are similar; however, disease progression following ingestion of pyrrolizidine alkaloid-containing plants is rarely reversible, and euthanasia is often required [112].

Across all cases, the most significant risk factor was inadequate grassland management, which resulted in increased coverage of poisonous plant species. As noted, poor pasture quality can elevate the risk of poisoning. In addition, animals spent prolonged periods exposed to direct sunlight during times of elevated UV index, which exacerbated phototoxic effects. Finally, limited farmer awareness of photosensitizing plants contributed to the outbreaks, as preventive measures were often unfamiliar or not implemented [108].

## 10. Conclusions

Secondary plant metabolites capable of inducing photosensitization may manifest as seasonal clinical syndromes in grazing livestock. The environmental and genotype-specific determinants governing the biosynthesis of light-absorbing bioactive molecules in plants remain incompletely understood. Nevertheless, such compounds occur in both cultivated forage species and invasive weeds, posing an ongoing risk to pasture-based production systems.

Within the animal organism, photosensitization represents a cascade of photodynamic reactions driven by light-absorbing molecules, typically heterocyclic or polyphenolic in nature. Primary photosensitization arises following ingestion or dermal exposure to photodynamic compounds, which increase cutaneous susceptibility to solar radiation. Clinical severity is heightened in animals with poorly pigmented or non-wool-covered skin, where natural photoprotection is limited.

Secondary (hepatogenous) photosensitization most commonly results from the accumulation of phylloerythrin due to impaired hepatic metabolism. Hepatotoxic plant compounds may induce severe liver damage, disrupting the normal excretion of chlorophyll degradation products from the bloodstream, thereby precipitating photosensitization in affected animals.

To reduce the severity of photosensitization, farmers should minimize direct sunlight exposure in affected animals by providing adequate shelter. Feeding management is also essential; grazing should be temporarily avoided and replaced with hay feeding [113,114], as hunger and thirst diminish the animals’ selective grazing capacity and increase the likelihood of ingesting toxic plants.

Grassland management represents another key preventive strategy. Farmers should isolate affected pasture areas and, where feasible, eliminate poisonous plant species or apply seed control measures to maintain high-quality swards [114]. However, these management and prevention practices are only effective if farmers and veterinarians are familiar with the local botanical flora. Therefore, targeted education and professional training are also crucial components of prevention programs [115].

As the mechanism of action underlying photosensitization has been extensively documented in the scientific literature, the primary priority for veterinary clinical practice is the identification of an increasing number of plant species capable of inducing photosensitization, a task that necessitates continued phytochemical investigation. The publication of detailed case reports enables veterinarians to select appropriate therapeutic interventions while also supporting the development of broader preventive strategies.

## Figures and Tables

**Table 1:** Comparative features of solar dermatitis and photosensitization in livestock.

Feature	Solar Dermatitis (Sunburn)	Photosensitization
etiology	direct ultraviolet (UV) radiation damage to skin	activation of photodynamic compounds by UV/visible light
primary cause	excess solar exposure, especially in unpigmented skin	ingestion or dermal exposure to photosensitizing agents or hepatic dysfunction with phylloerythrin accumulation
pathogenesis/mechanism	direct UV-induced cellular injury DNA damageinflammation	light activation of photodynamic molecules → reactive oxygen species (^1^O_2_, ROS) → oxidative tissue injury
role of liver	no hepatic involvement	none in primary form; central in secondary form
lesion distribution	restricted to sun-exposed, non-pigmented, sparsely haired areas	similar distribution, but may be more severe and widespread
systemic signs	typically absent	present in hepatogenous form: depression, icterus, weight loss, hepatic failure signs
histopathology (skin)	epidermal necrosis, keratinocyte apoptosis, superficial inflammation	necrotizing dermatitis, epidermal hyperplasia, severe phototoxic injury
prognosis	generally favorable with sun avoidance	variable: good in primary if exposure stops, guarded to poor in hepatogenous cases
prevention	shade, pigmentation, UV protection	pasture management, toxic plant control, liver health monitoring

## Data Availability

No new data were created or analyzed in this study. Data sharing is not applicable to this article.

## Abbreviations

The following abbreviations are used in this manuscript:

AST	aspartate aminotransferase
GGT	gamma-glutamyl transferase
PA	pyrrolizidine alkaloids
ROS	reactive oxygen species
UV	ultraviolet
SC	stratum corneum
SB	stratum basale
SPP	secondary plant products/metabolites
